# Impact of Push Notifications on Physical Activity and Sodium Intake Among Patients with Hypertension: Microrandomized Trial of a Just-in-Time Adaptive Intervention

**DOI:** 10.2196/78218

**Published:** 2026-01-07

**Authors:** Jessica R Golbus, Michael P Dorsch, Yuxuan Chen, Tanima Basu, Evan Luff, Predrag Klasnja, Mark W Newman, Lesli E Skolarus, Walter Dempsey, Brahmajee K Nallamothu

**Affiliations:** 1 Division of Cardiovascular Medicine, Department of Internal Medicine, University of Michigan, 2723 Cardiovascular Center, 1500 E Medical Center Dr. SPC 5853, Ann Arbor, MI, 48109, United States, 1 7342325045; 2 Michigan Integrated Center for Health Analytics and Medical Prediction (MiCHAMP), University of Michigan, Ann Arbor, MI, United States; 3 Department of Clinical Pharmacy, College of Pharmacy, University of Michigan, Ann Arbor, MI, United States; 4 Department of Biostatistics, University of Michigan School of Public Health, Ann Arbor, MI, United States; 5 School of Information and Department of Electrical Engineering and Computer Science, University of Michigan, Ann Arbor, MI, United States; 6 Division of Stroke Vascular Neurology, Davee Department of Neurology, Northwestern University Feinberg School of Medicine, Chicago, IL, United States

**Keywords:** digital health, hypertension, mHealth, mobile health, physical activity, just-in-time adaptive intervention

## Abstract

**Background:**

Achieving adequate blood pressure control is challenging for patients and clinicians. Digital hypertension management solutions that use push notifications to promote lifestyle management have been proposed as an approach, but their effectiveness remains unknown.

**Objective:**

This analysis was designed to interrogate the independent and short-term effects of push notifications, tailored to participant and environmental factors, and on physical activity levels and sodium intake among individuals with hypertension.

**Methods:**

The myBPmyLife study was a 6-month randomized controlled trial of participants with self-reported hypertension recruited from an academic medical center and federally qualified health centers. A core component of the intervention consisted of microrandomized push notifications promoting lifestyle modifications that were randomly delivered at 4 daily time points and focused on physical activity and dietary sodium intake. Our primary outcome for this secondary analysis was the step count 60 minutes after a physical activity notification and lower-sodium food choices 24 hours after a dietary notification. This analysis focuses on the results of the microrandomized trial and used a centered and weighted least squares method adapted for 2 or more treatments.

**Results:**

A total of 298 participants were randomized to the intervention arm, of whom 287 had data available for analysis. Participants’ mean age was 59.5 (SD 13.1) years, 138 (48.1%) were women, and 206 (71.8%) were White. Participants were randomized at 187,517 time points over 6 months, which led to 0.96 (SD 0.86) push notifications per day divided between activity (50.4%; SD 0.4) and dietary (49.8%; SD 0.4) notifications. Activity notifications did not increase step count in the 60 minutes after a notification (estimate 1.01, 95% CI 0.98‐1.04; *P*=.40). Similarly, dietary notifications did not impact the number of lower-sodium food choices in the subsequent 24 hours (estimate 0.93, 95% CI 0.83‐1.04; *P*=.23), but in exploratory post hoc analyses, did increase mobile app use by 95.5% (95% CI 1.81‐2.10; *P*<.001), mobile app clicks or searches by 93.7% (95% CI 1.72%‐2.16%; *P*<.001), and low sodium searches by 113.0% (95% CI 1.73‐2.53; *P*<.001), all within 60 minutes.

**Conclusions:**

In patients with hypertension, push notifications did not impact short-term physical activity levels or dietary sodium intake but did improve intervention engagement.

## Introduction

Nearly half of all US adults have hypertension, though only 1 in 4 have their blood pressure (BP) adequately treated [1]. Achieving adequate BP control is challenging for patients and clinicians given the episodic nature of clinical encounters, high patient volumes, and the silent nature of the disease process, promoting clinical inertia [2]. Digital hypertension management solutions aim to improve BP control by promoting patient self-management and expanded access to care. These solutions often center around connected BP cuffs for self-monitoring, with or without medication management, and may be paired with behavior and lifestyle change interventions in the form of mobile apps, text messages or push notifications, and phone calls [2]. Although these methods could enhance BP control, the effectiveness of each digital intervention component—especially within a multicomponent framework—continues to be a significant and unresolved issue.

We recently completed the myBPmyLife study, which evaluated a mobile health (mHealth) intervention designed to improve BP control by promoting increased physical activity and the selection of lower-sodium food choices. The study recruited participants from an academic medical center and federally qualified health centers [3]. The intervention consisted of a mobile app designed to facilitate goal setting and feedback and contextually tailored push notifications delivered as a part of a microrandomized trial, a novel experimental design in which participants are serially randomized to different types or levels of an intervention (eg, push notifications) [4]. While the intervention reduced sodium intake and improved physical activity levels, it failed to lower systolic BP (SBP) compared to a control group that engaged in BP self-monitoring alone [5].

One important question that remained was whether push notifications led to immediate short-term effects or mediated behavior change that occurred on a longer timescale. To address this question, we present the myBPmyLife study’s 6-month microrandomized trial results, which focuses on important secondary, mechanistic outcomes of this trial. This analysis was designed to interrogate the independent and short-term effects of push notifications, tailored to participant and environmental factors, on physical activity levels and sodium intake. Such an approach allows us to isolate the causal effects of push notifications relative to the larger intervention package. We hypothesized that tailored push notifications would increase participants’ step counts in the 60 minutes following delivery and decrease sodium intake in the subsequent 24 hours.

## Methods

### Study Design and Participants

The myBPmyLife study was a prospective, remotely administered, randomized-controlled trial (ClinicalTrials.gov NCT05154929) of 602 participants with self-reported hypertension (CONSORT [Consolidated Standards of Reporting Trials] guidelines; Checklist 1). Participants were recruited from the University of Michigan Health and the Hamilton Community Health Network, a series of federally qualified health centers in Flint, Michigan. The analysis described here focuses on the results of the microrandomized trial, which was embedded within the intervention arm of the randomized controlled trial, and which was composed of push notifications promoting increased physical activity and the selection of lower-sodium food choices. The data and code that support this study are openly available through GitHub.

Participants were eligible for the study if they were 18 years or older with self-reported hypertension, had no changes in their antihypertensive therapies in the preceding 4 weeks (if on medical therapies), consumed >1500 mg of daily sodium, and owned a compatible smartphone. Sodium intake was assessed by the Block Sodium Screener [6] after informed consent was obtained, and those who consumed <1500 mg/d of sodium were ineligible. Participants with contraindications to physical activity or to following a low-sodium diet were excluded from the study. The full inclusion and exclusion criteria have been previously published [3].

### Ethical Considerations

The study was approved by the University of Michigan Health IRB (HUM00205845). All participants participated in an informed consent process and signed consent forms. All study data are deidentified and remain anonymous. Participants received up to US $100 over 6 months for their time completing study tasks. All participants were provided with a Bluetooth-connected smartwatch (Fitbit Versa 2) and a Bluetooth BP monitor (Omron Evolv BP7000) for the purposes of study participation.

### Study Procedures

The myBPmyLife study was conducted between December 2021 and July 2023. Study procedures have been previously published. Briefly, participants underwent remote screening, recruitment, and consent processes. Consent forms were signed using the mobile study app myDataHelps (CareEvolution, LLC). Following the completion of the Block Sodium Screener, eligible participants were randomized 1:1 to the intervention arm, which received a BP monitor and an mHealth intervention delivered through a mobile app and a smartwatch, or to the enhanced usual arm, which received a BP monitor and smartwatch alone. Randomization schemes were created by study statisticians in Randomize.net and performed by study staff using a permuted block design with variable block sizes of 2-6, stratified by study site. Researchers and participants were unblinded to allocation assignment given the nature of the intervention.

Following informed consent, participants in both study arms were mailed a Fitbit Versa 2 and a Bluetooth BP monitor (Omron Evolv BP7000) and provided instructions on downloading the associated mobile apps to enable data sharing. All participants subsequently underwent remote enrollment appointments, at which time they were assisted with pairing their smartwatches and smartphones as needed. Participants randomized to the intervention arm additionally answered a series of questions to facilitate intervention tailoring, including those on mobility, confidence in selecting lower-sodium food choices, preferred name, times of day for push notification (ie, morning, lunch, afternoon, and evening times), and preferred day for grocery shopping. These preferences could be changed during the study upon participant request. Following enrollment but before intervention receipt, participants experienced a baseline period in which no interventions were delivered to establish baseline step counts.

### Description of the Intervention

The myBPmyLife study was designed to increase physical activity levels and promote the selection of lower-sodium food choices through an mHealth intervention grounded in the behavior change strategies of goal setting, prompts, visualizations, and feedback. The intervention consisted of both static (ie, mobile app with a central visualization promoting strategic feedback and goal setting) and dynamic components, with the latter referring to push notifications delivered as part of the microrandomized trial [5]. Microrandomized trials are an experimental approach to guide the design of just-in-time adaptive interventions. Just-in-time interventions, within the context of health behavior change, aim to provide support at times of opportunity or risk when individuals are receptive to change [7]. As such, just-in-time interventions aim to shape behavior change both in the moment and over time.

Microrandomized trials are an experimental design in which participants are serially randomized to receive (or not receive) different types or levels of an intervention (eg, push notifications) at different time points (ie, decision points) over the length of a study [4]. Intervention efficacy is ascertained by 1 or more proximal outcomes, which refer to short-term outcomes hypothesized to mediate a desired long-term effect. By leveraging intraindividual contrasts, microrandomization is a powerful experimental design for determining causal effects. In the myBPmyLife trial, microrandomized push notifications comprised a core component of the intervention (Figure S1 in Multimedia Appendix 1). They were designed to promote low-level physical activity and the selection of lower-sodium food choices. Activity notifications were tailored based on the time of day (ie, morning, lunch, afternoon, and evening), day of week (ie, weekend vs weekday), weather, and mobility. Similarly, dietary notifications were tailored according to the time of day, day of the week (ie, weekend, weekday, or grocery day), and participants’ confidence in selecting lower-sodium food choices. Both notification types consisted of expert-generated and community-generated notifications, with the latter tailored based on participants’ site of enrollment (ie, University of Michigan Health or Hamilton Community Health Network). A subset of notifications was personalized using participants’ preferred names [8].

The study was designed so that participants would receive 1 activity or dietary notification per day, on average, throughout the study. Thus, participants had a 25% probability of receiving a notification at each decision point, divided equally between activity and dietary notifications. The proximal outcomes for the microrandomized trial serve as key secondary outcomes from the overarching clinical trial. The proximal outcome for activity notifications was step count 60 minutes after a decision point as determined by the smartwatch or mobile phone, as these messages were intended to be actionable in real time. For dietary notifications, the proximal outcome was self-reported lower-sodium food choices in the mobile app within 24 hours of a decision point, as these messages were intended to be applicable at a future time around meals or snacks and when at restaurants or grocery stores. For the dietary analysis, the decision was made post hoc to explore a series of more proximal engagement measures, as dietary notifications encouraged participants to use the mobile app to identify lower-sodium alternatives to commonly consumed or purchased foods. These exploratory outcomes included mobile app use (yes or no), the number of mobile app searches or clicks, and the number of low salt searches within the mobile app, all within 60 minutes of a decision point.

### Statistical Analysis

Baseline clinical characteristics are described as means and SD for continuous symmetric variables and median with IQR for skewed continuous variables. Categorical variables are presented as counts and percentages. We performed Student 2-tailed *t* tests for bivariate comparisons between continuous variables and chi-square tests for comparisons across categorical variables.

For both the physical activity and dietary analyses, the intervention period was defined as the time of participants’ first decision point at or beyond day 8 and lasting until day 180 (ie, maximum study period of 173 d). This was defined to account for variability in the duration of the baseline period (as determined by smartwatch wear time) and in the completion of the final surveys.

For the physical activity analysis, we evaluated the effect of delivering an activity notification compared to no notification on step count 60 minutes after a decision point. As step counts were positively skewed, 0.5 was added to all counts before natural log transformation. Analyses used a version of the weighted and centered least squares (WCLS) method. The WCLS approach is designed for microrandomized trials, where treatments occur frequently and moderators may be affected by prior interventions. This method involves centering time-varying treatments using their conditional means based on past history and applying weights similar to inverse probability of treatment weighting in causal inference. By combining centering and weighting, WCLS offers robustness against the misspecification of the working model of weighted outcome condition on history [9]. Our analyses used a version of the WCLS method adapted to account for 2 or more treatments [9]. Such an approach increases statistical power by avoiding treatment-specific models and guarantees the robust, unbiased inclusion of covariates to reduce noise. Models were adjusted for age (<65 vs >65 y), gender, race (White vs non-White), baseline mean daily step count (dichotomized on mean value), step count 30 minutes before a decision point (standardized as mean or SD), and time (d). At each decision point, participants were considered available to receive the intervention if they were wearing their smartwatches consistent with prior studies [10]. This was operationalized by requiring that participants have at least 1 heart rate measurement recorded on their smartwatches in the 30 minutes before a decision point. Models included decision points at which participants were available to receive notifications, and step count data were available in the 30 minutes before and 60 minutes after a decision point (ie, excluded 5807, 3.1% of decision points). Subsequently, we conducted a series of exploratory analyses to understand the impact of key demographic (ie, age, gender, and community) and clinical (ie, baseline step count) characteristics on our primary outcome by evaluating the interaction between these covariates and treatment. An additional exploratory model evaluated the impact between time as a quadratic term and treatment. Finally, as outcome data can be present in instances of missing wear time as data can come from both the mobile phone and smartwatch, we conducted a sensitivity analysis in which we excluded the data from participants with zero or missing wear time 3 hours after a decision point. This allowed us to disentangle instances of smartwatch nonwear from zero step counts.

For the dietary analysis, we evaluated the effect of delivering a dietary notification compared to the composite of an activity notification or no notification on lower-sodium choices (as denoted in the mobile app) within 24 hours of a decision point. Given the potential overlap in push notifications over 24 hours, the assessment of treatment effect included only decision points at which participants received a dietary notification, and no additional dietary notifications were sent in the subsequent 24 hours (excluded 42,313, 22.6% decision points). These were contrasted to decision points at which no dietary notification was sent at that time or in the subsequent 24 hours. As lower-sodium choices were zero-inflated, we used the method proposed by Liu et al [11] and used the R (R Foundation for Statistical Computing) function *emee* in package *MRTAnalysis*. Models were adjusted for age (<65 vs >65 y), gender, race (White vs non-White), baseline sodium intake as measured by the Block Sodium Screener (dichotomized on mean value), low salt choices 30 minutes before a decision point, and time (d). Participants were considered available at all decision points given the extended time window for outcome assessment. As with the physical activity analysis, we also conducted a series of exploratory analyses to understand the potential impact of key demographics (ie, age, gender, and community) and baseline characteristics (eg, baseline sodium intake) on sodium intake by evaluating the interaction between these covariates and treatment. An additional exploratory model evaluated the impact between time as a quadratic term and treatment. Similar models were created for our exploratory outcomes of mobile app use (yes or no), number of mobile app clicks or searches, and number of low sodium searches within the mobile app, all within 60 minutes of a decision point. These similarly used the method proposed by Liu et al [11], though it included all decision points (N=187,517), unlike the prior analysis. Covariates in these models included age (<65 vs >65 y), gender, race (White vs Non-white), and either mobile app use, number of search or clicks, or number of low sodium choices 30 minutes before a decision point, respectively. In all cases, *P*<.05 was considered statistically significant. All analyses were conducted in R (version 4.3.1; R Foundation for Statistical Computing).

## Results

### Study Population

**Figure 1.**
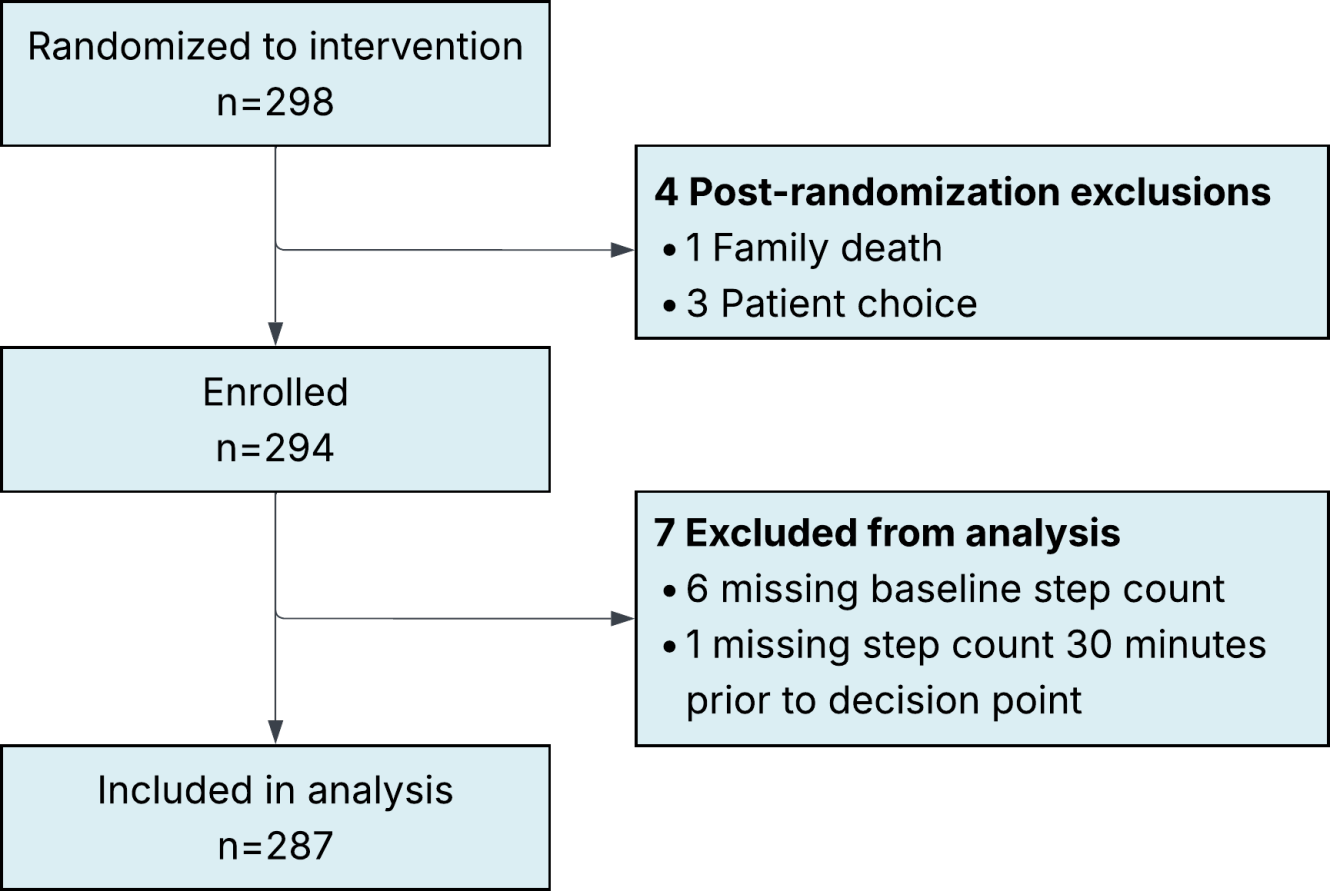
CONSORT (Consolidated Standards of Reporting Trials) diagram. Between December 2021 and July 2023, 912 participants were screened for eligibility. Following informed consent, participants were randomized 1:1 to the intervention and enhanced usual care arms with 298 participants randomized to the intervention arm of the trial. Of these, 4 participants withdrew before intervention receipt (postrandomization exclusions), and an additional 15 participants withdrew from the study but allowed their data to be used up until their withdrawal date. A total of 287 participants were included in the analysis of the microrandomized trial.

Between December 2021 and July 2023, 912 participants were screened for eligibility, of whom 602 were ultimately enrolled. A total of 298 participants were randomized to the intervention arm of the study, 4 of whom withdrew before intervention receipt during the initial 5 days of the study (Figure 1). An additional 15 participants withdrew from the study, though they allowed their data to be used up until their withdrawal date (Figure 1). Post hoc, an additional 7 participants were excluded from the analysis (6 due to missing baseline step count and 1 missing step count 30 min prior to the decision point), leaving 287 participants available for the primary analysis.

Participants were enrolled in the study for a median of 180 (IQR 37‐180) days with a median intervention duration of 171 (IQR 25‐173) days. The baseline characteristics of the population are displayed in Table 1. Participants had a mean age of 59.5 (SD 13.1) years, 138 (48.1%) were women, and 206 (71.8%) were White. Most were from the University of Michigan Health (242/287, 84.3%). Participants' baseline step count was 7408.1 (SD 3611.9) steps per day, and baseline sodium intake was 3072.7 (SD 1049.9) mg/d.

**Table 1.** Demographic and clinical characteristics of study population (N=287).

Characteristics	Ann Arbor (n=242)	Flint (n=45)	Overall
Age (y), mean (SD)	61.35 (12.5)	49.24 (11.6)	59.5 (13.1)
Gender, n (%)			
Woman	106 (43.8)	32 (71.1)	138 (48.1)
Man	136 (56.2)	13 (28.9)	149 (51.9)
Race, n (%)			
Asian	28 (11.6)	0 (0)	28 (9.8)
Black	20 (8.3)	20 (44.4)	40 (13.9)
Multiple	2 (0.8)	4 (8.9)	6 (2.1)
Other^a^	5 (2.1)	2 (4.4)	7 (2.4)
White	187 (77.3)	19 (42.2)	206 (71.8)
Ethnicity, n (%)			
Hispanic	11 (4.5)	1 (2.2)	12 (4.2)
Non-Hispanic	231 (95.5)	44 (97.8)	275 (95.8)
Baseline step count (steps/d) mean (SD)	7633.09 (3564.1)	6197.87 (3666.5)	7408.06 (3611.9)
Baseline estimated dietary sodium intake (mg/d), mean (SD)	3054.95 (1016.0)	3167.96 (1223.4)	3072.67 (1049.7)
Self-reported comorbid conditions, n (%)			
Chronic kidney disease	12 (5.0)	4 (8.9)	16 (5.6)
High cholesterol	109 (45.0)	16 (35.6)	125 (43.6)
Depression	39 (16.1)	13 (28.9)	52 (18.1)
Coronary artery disease	9 (3.7)	1 (2.2)	10 (3.5)
Stroke	7 (2.9)	2 (4.4)	9 (3.1)
Diabetes mellitus	57 (23.6)	9 (20.0)	66 (23.0)

a Other race: American Indian, Native Hawaiian or Other Pacific Islander, or Other or refused to answer race question

### Study Execution

Over the duration of the intervention, participants were randomized to receive or not receive a push notification at 187,517 decision points (ie, time points; median 679, IQR 89‐694 decision points per participant). On average, participants had 4.0 (SD 0.2) decision points per day and were randomized to receive a push notification at 24.9% (SD 0.2) of decision points. These were nearly equally split between activity notifications (50.4%; SD 0.4) and dietary notifications (49.8%; SD 0.4), consistent with the study design. On average, participants were randomized to receive 0.96 (SD 0.86) push notifications per day, distributed nearly equally over the 4 time points (Tables S1-S3 in Multimedia Appendix 1). After accounting for participants’ availability (considered available at mean 81.3%, SD 0.3 of decision points), participants were presumed to have received 0.79 (SD 0.83) push notifications each day.

### Activity Intervention

**Figure 2.**
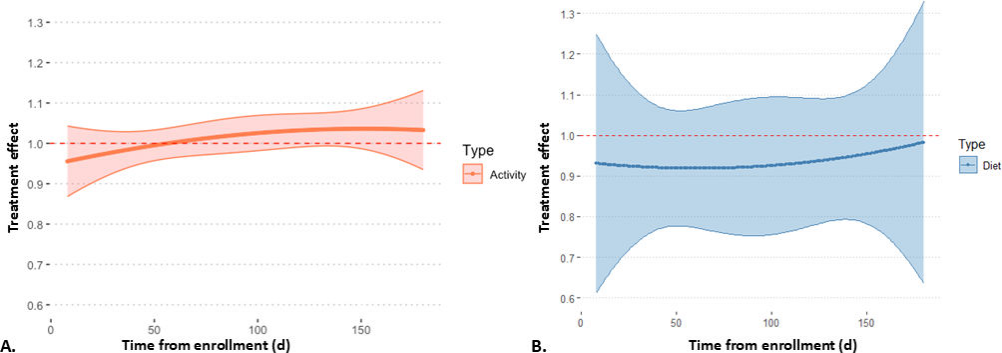
Impact of receiving (A) an activity notification on step count 60 minutes after a decision point or (B) a dietary notification on lower-sodium food choices 24 hours after a decision point. The models show treatment effect over time, with time modeled as a quadratic term. Activity notifications and dietary notifications did not significantly impact step count or the number of lower-sodium food choices, respectively. The results were exponentiated for interpretability.

The mean step count in the 60 minutes following a decision point was 456.1 (SD 707.0) steps. In a multivariable model accounting for the demographic and baseline characteristics of participants, activity notifications did not significantly impact 60-minute step count (estimate 1.01, 95% CI 0.98‐1.04; *P*=.40; Tables S4 and S5 in Multimedia Appendix 1; Figure 2). In a subsequent sensitivity analysis excluding decision points at which participants had zero hour or missing wear time in the 3 hours after a decision point (17,452, 9.6% of decision points), the results were similar with no significant change in 60-minute step count (Table S6 in Multimedia Appendix 1). To understand potential time-varying effects, we performed an exploratory analysis in which we modeled the intervention effect as a quadratic term over time, which did not significantly impact intervention efficacy (Figure 2). Similarly, in a sequence of univariable moderator analyses, age, community, and gender did not significantly impact intervention efficacy. In an exploratory analysis, however, push notifications were more effective in the subgroup of participants that were less active at baseline (dichotomized based on mean daily step count), increasing 60-minute step count by 4% (estimate 1.04, 95% CI 1.00‐1.08; *P*=.03).

### Dietary Intervention

In a multivariable model accounting for demographic and baseline characteristics, dietary notifications did not change the number of lower-sodium food choices in the 24 hours after a decision point (estimate 0.93, 95% CI 0.83‐1.04; *P*=.23; Tables S4 and S7 in Multimedia Appendix 1; Figure 2). The results were similar in all subgroups of the population and did not change over time.

**Figure 3.**
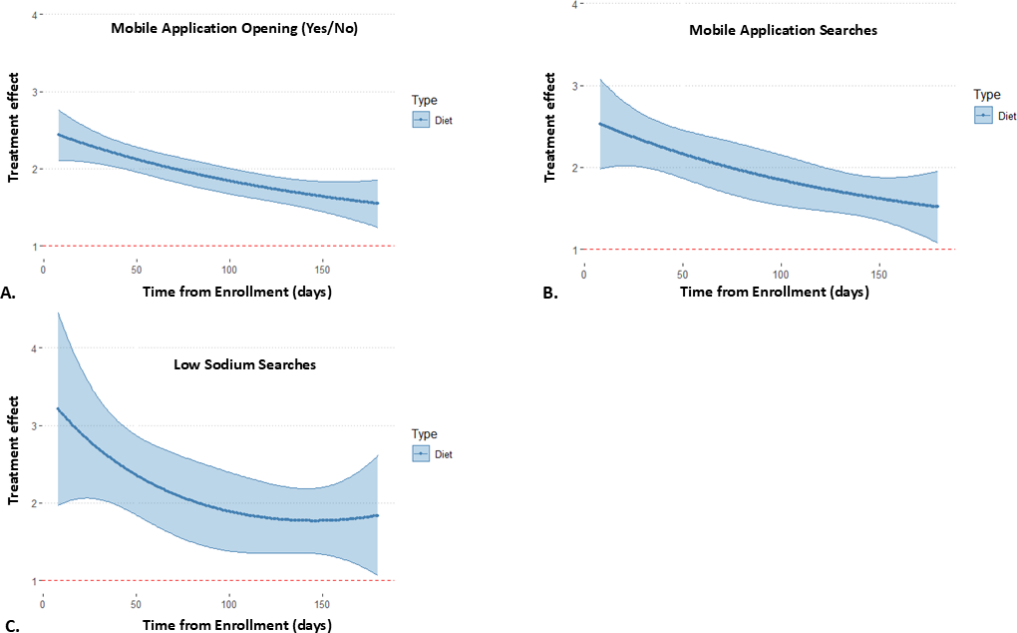
Intervention engagement after dietary notifications. Dietary notifications significantly increased (**A**) mobile app use, (**B**) number of mobile app searches/clicks, and (**C**) number of low sodium searches, all within 60 minutes of a decision point. The models show treatment effect over time, with time modeled as a quadratic term. The results were exponentiated for interpretability.

Subsequently, we conducted an exploratory analysis to evaluate the impact of push notifications on immediate measures of intervention engagement. Dietary notifications increased mobile app use in the subsequent 60 minutes by 95.5% (estimate 1.96, 95% CI 1.81‐2.10; *P*< .001; Figure 3). Similarly, dietary push notifications increased the number of mobile app clicks or searches by 93.7% (estimate 1.94, 95% CI 1.72%‐2.16%; *P*<.001) and increased the number of low sodium food searches by 113% (estimate 2.13, 95% CI 1.73‐2.53; *P*<.001), 60 minutes after a decision point (Figure 3). In all cases, the effect of dietary push notifications was the highest at the beginning of the study and decreased over time, though it remained significant throughout the study period.

## Discussion

### Principal Findings

Digital hypertension solutions, typically delivered as multicomponent interventions, have shown promise for enhancing BP control [2,12,13]. However, the independent effects of push notifications when offered as part of a comprehensive digital hypertension intervention remain unknown. This is especially true with regard to the impact of push notifications on behavior change. We found through this trial that push notifications, tailored to participant (eg, community, mobility, and time) and environmental (eg, weather) factors, did not increase short-term physical activity levels or reduce sodium intake despite improving overall measures of these outcomes at 6 months. In an exploratory analysis, however, push notifications promoting physical activity were more effective for less active individuals, though this effect was overall very small, and for the overall cohort, the intervention was insufficient to overcome a participant’s existing behavioral inertia. We also identified through an exploratory analysis that notifications that encouraged participants to interact with the mobile app and select lower-sodium food choices led to a nearly 2-fold increase in mobile app engagement, a measure of mechanical engagement though not necessarily effective behavioral engagement. While this effect persisted over the 6-month study period, the magnitude of effect decremented over time, suggesting either internalization of the behavior of interest (ie, engagement with the mobile app was no longer necessary for promoting adherence to a lower-sodium diet) or habituation.

Interpreting these results is best done within the context of those from the larger randomized controlled trial in which we found that the digital intervention package, consisting of both a mobile app and push notifications, increased physical activity levels (mean difference 489 steps, 95% CI 22-956) and reduced sodium intake (mean difference –285 mg, 95% CI −462 to –108) at 6 months [5]. One possible explanation for these results is that the interaction with the mobile app, which included multiple behavior change techniques (ie, goal setting, self-monitoring, and feedback) but not necessarily the content of the push notifications themselves, mediated the observed effects. This is supported by prior literature demonstrating positive associations between engagement with digital health interventions and health outcomes [14-16]. An alternative explanation, however, would be that push notifications did mediate the observed results, but that the proximal outcomes did not adequately capture their long-term impact. This could be due to lagged effects (ie, increase in physical activity beyond 60 min), incompletely captured effects (ie, failure to record lower-sodium food choices within the mobile app), or slower-developing mediators of the distal outcome (ie, knowledge about lower-sodium food choices, salience of activity goals). Future interventions should consider incorporating both proximal and intermediate outcomes within their study design, with the latter potentially capturing lagged effects (eg, 24 h step count and lower-sodium food choices over 48 h) or more slowly developing mediators of the target behavior.

### Comparison With Prior Work

In general, digital hypertension solutions have focused on promoting BP self-monitoring, medication management, and lifestyle modification [2]. However, a challenge within the field has been in understanding the relative contributions of different digital intervention components, particularly when delivered as 1 part of a comprehensive intervention. In the pragmatic, randomized controlled trial BP Home, for example, over 2000 patients were randomized to BP self-monitoring using a standard device or to enhanced self-monitoring using a connected smartphone app [17]. Both groups experienced similarly large reductions in SBP (>10 mm Hg), without significant differences between the 2 groups. A second study found no significant differences in SBP reduction at 6 months using an artificial intelligence–enhanced conversational smartphone app promoting self-management, BP measurement, and lifestyle management, compared to a regular smartphone app paired with a home BP monitor [18]. There was, however, greater self-confidence in controlling BP in the intervention group and a trend toward greater self-reported physical activity.

To disentangle the effects of varying intervention components, Tucker et al [12] conducted an individual participant meta-analysis with over 7000 patients from 15 studies. Self-monitoring was associated with reduced SBP at 12 months, though this effect was strongly influenced by the intensity of co-interventions, with no effect with self-monitoring alone and a greater effect when combined with more intensive interventions, such as lifestyle counseling or systematic medication titration. These results have been supported by those from other studies, which, while mixed, suggest in aggregate that successful interventions are those with more comprehensive functionality and that incorporate medication management in conjunction with a care team [2,13]. A limitation of these and other studies, however, has been their focus on evaluating the distal effects of an intervention package, often delivered through a mobile app with multiple behavioral components. Our microrandomized trial analysis overcomes these limitations by isolating the impact of push notifications on shorter-term mediators of the desired long-term effect (eg, lifestyle management) and suggests how the mobile intervention package may impact long-term behavior change.

### Study Strengths

Our study has several strengths. First, this is one of the first microrandomized trials that we are aware of among individuals with hypertension. Microrandomized trials are a novel experimental design, and as such, few studies have been performed among individuals with cardiovascular disease or cardiovascular risk factors. By incorporating serial randomization, microrandomized trials can provide insight into the causal effects of an intervention component over time in such patients. This serves to advance the science of behavioral interventions, which have traditionally been optimized using a series of randomized controlled trials. Randomized controlled trials, however, are designed to assess the average effect of an intervention package on a behavior of interest and not to investigate which components of an intervention are most efficacious, their time-varying effects, or what psychosocial or contextual factors impact their efficacy [4,19]. Second, we delivered the trial remotely and enrolled participants from 2 sites, including a series of federally qualified health centers, enhancing the diversity of the study population and the generalizability of our findings. In general, the data around mHealth interventions for individuals with hypertension with digital barriers or from underrepresented groups have been limited [20]. Finally, we followed participants for 6 months, addressing a critical limitation of many mHealth interventions, namely the short time horizon.

### Limitations

This study should be interpreted within the context of its limitations, which relate both to the study population and the intervention design and analysis. First, we only enrolled participants with a compatible smartphone. Smartphone ownership, however, is common across age, race, and socioeconomic groups, and we ensured the use of a broad range of possible devices [21]. Second, participants were active at baseline (mean 7408 [SD 3611.9] steps per day), had only mildly elevated BP, and were connected to their health care system. Given that this population was already active at baseline, this may have led to a ceiling effect. Similarly, the intervention may have been perceived as less salient to participants with relatively well-controlled BPs. Whether the results would be similar with less active individuals or to those with poorly controlled BP is unknown. Third, push notifications were tailored on a limited number of environmental and participant-level factors. It is unknown whether notifications would be more effective in promoting lifestyle modification if tailored to psychosocial constructs or an extended set of participant factors (eg, recent physical activity). We also did not evaluate notification efficacy based on message framing, nor did we evaluate treatment moderation based on attributes of the notifications (eg, time of day, weather). For example, in a 2×2 randomized experiment of over 500 participants designed to evaluate message framings, participants preferred the ability to choose message framing (autonomy-supportive vs controlling language) over the presumed preference for autonomy-supportive language [22]. Future studies should consider an expanded set of tailoring variables and evaluate for treatment moderation by the characteristics of the notifications. Fourth, we required that participants self-report low sodium choices within the mobile app. It is thus possible that dietary notifications increased the number of low sodium food choices, though these were not reported by participants in the mobile app. Fifth, it is possible that the effect size of notifications may have been less than anticipated, leading us to underpower the study for our proximal outcomes. Finally, we required that participants be wearing their smartwatches to be considered available for the physical activity intervention. We assumed that participants were wearing their smartwatches if they had 1 or more heart rate measurements in the 30 minutes before a message was sent. Although this increased the rigor of our analyses, we cannot confirm that participants were wearing their smartwatches when push notifications were sent. It is also possible that push notifications had a delayed or unmeasured effect for participants not wearing their smartwatches or who received the push notifications at a later time.

### Conclusions and Future Directions

In conclusion, in this microrandomized trial, we demonstrated that tailored push notifications did not increase short-term physical activity levels or reduce sodium intake among individuals with hypertension. We did observe greater mobile app engagement following push notifications, which may have mediated the observed longer-term effects on sodium intake and physical activity levels. These results suggest that push notifications may be effective in promoting intervention engagement. Additional studies, however, are needed to identify which individuals benefit most from push notifications and how to optimally tailor push notifications based on environmental and psychosocial factors, particularly when delivered as part of multicomponent interventions. Furthermore, future studies may consider mediation analyses to enhance our understanding of the impact of intervention engagement on longer-term measures of behavior change and which evaluate the impact of novel methodological approaches such as reinforcement learning algorithms on notification efficacy.

## Supplementary material

10.2196/78218 Multimedia Appendix 1 Supplemental tables and figures.

10.2196/78218 Checklist 1 CONSORT 2025 checklist.

## Abbreviations

BP: blood pressure
CONSORT: Consolidated Standards of Reporting Trials
mHealth: mobile health
SBP: systolic blood pressure
WCLS: weighted and centered least square
