# Supplementary material for: Impact of Push Notifications on Physical Activity and Sodium Intake Among Patients with Hypertension: Microrandomized Trial of a Just-in-Time Adaptive Intervention
Source: J Med Internet Res. 2026 Jan 7;28:e78218. doi: 10.2196/78218 (PMC12779098; doi:10.2196/78218)
Supplement: Multimedia Appendix 1 [file jmir-v28-e78218-s001.docx]

**SUPPLEMENTAL MATERIAL**

**Impact of Push Notifications on Physical Activity and Sodium Intake Amongst Patients with Hypertension:**

**A Micro-Randomized Trial of a Just-In-Time Adaptive Intervention**

Jessica R. Golbus, MD, MS^1,2^*, Michael P. Dorsch, PharmD, MS^3*^, Yuxuan Chen^4^, Tanima Basu, MA, MS^1^, Evan Luff, MS^1^, Predrag Klasnja, PhD^5^, Mark W. Newman, PhD^5^, Lesli E. Skolarus MD, MS^6^, Walter Dempsey PhD^4^, Brahmajee K. Nallamothu MD, MPH ^1,2 ,7^

*Co-first authors

1. Division of Cardiovascular Medicine, Department of Internal Medicine, University of Michigan, Ann Arbor, MI
2. Michigan Integrated Center for Health Analytics and Medical Prediction (MiCHAMP), University of Michigan, Ann Arbor, MI
3. Department of Clinical Pharmacy, College of Pharmacy, University of Michigan, Ann Arbor, MI
4. Department of Biostatistics, University of Michigan School of Public Health, Ann Arbor, MI
5. School of Information and Department of Electrical Engineering and Computer Science, University of Michigan, Ann Arbor, MI
6. Division of Stroke Vascular Neurology, Davee Department of Neurology, Northwestern University Feinberg School of Medicine, Chicago, IL
7. The Center for Clinical Management and Research, Ann Arbor VA Medical Center, Ann Arbor, MI

**Corresponding Author**

Jessica R. Golbus MD, MS

Department of Internal Medicine

Division of Cardiovascular Medicine

2723 Cardiovascular Center

1500 E. Medical Center Dr., SPC 5853

Ann Arbor, Michigan, 48109-5853

Phone: 734-243-5045

Email: [jgolbus@med.umich.edu](mailto:jgolbus@med.umich.edu)

**Table of Contents**

Page 3-8: Supplemental Table 1-7

Page 9: Supplemental Figure 1

| **Notification Number** | **N, days** | **Proportion of Notifications** |
| --- | --- | --- |
| 0 | 15669 | 33.1% |
| 1 | 20031 | 42.4% |
| 2 | 9436 | 20.0% |
| 3 | 1981 | 4.2% |
| 4 | 180 | 0.4% |

**Supplemental Table 1: Notifications per Day over Study Period.** Participants had a 25% probability of receiving a notification at each of 4 daily decision points. Displayed are the number of days at which participants received a given number of notifications (0-4) and the proportion of study days at which participants received each notification number.

|  | **Daily** | **Morning** | **Lunch** | **Afternoon** | **Evening** |
| --- | --- | --- | --- | --- | --- |
| **Overall** | 0.96 (0.86) | 0.24 (0.43) | 0.25 (0.43) | 0.25 (0.43) | 0.23 (0.42) |

**Supplemental Table 2: Mean daily notification number by time of day.** Displayed as mean with standard deviation.

|  | **Daily** | **Morning** | **Lunch** | **Afternoon** | **Evening** |
| --- | --- | --- | --- | --- | --- |
| **Activity notifications** | 0.50 (0.45) | 0.50 (0.50) | 0.51 (0.50) | 0.50 (0.50) | 0.50 (0.50) |
| **Dietary notifications** | 0.50 (0.45) | 0.50 (0.50) | 0.49 (0.50) | 0.50 (0.50) | 0.51 (0.50) |

**Supplemental Table 3: Notification proportion by time of day and notification type.** Displayed as mean with standard deviation.

|  | **Estimate** | **95% Confidence Interval** | **P-value** |
| --- | --- | --- | --- |
| ***Step count, 60 minutes*** | | | |
| **Activity notification** | 1.01 | 0.98 – 1.04 | .40 |
| ***Lower sodium food choices, 24 hours*** | | | |
| **Dietary notification** | 0.93 | 0.83 – 1.04 | .23 |

**Supplemental Table 4: Impact of receiving an activity notification on step count 60 minutes after a decision point (top) or a dietary notification on lower sodium food choices 24 hours after a decision point (bottom).** In a multivariable model accounting for demographic and baseline characteristics of participants, activity notifications did not significantly change step count in the 60-minutes after a decision point (top) and dietary notifications did not change the number of lower sodium food choices in the 24 hours after a decision point (bottom).

|  | **Estimate** | **95% Confidence Interval** | **P-value** |
| --- | --- | --- | --- |
| Activity notification | 1.01 | 0.98 – 1.04 | 0.40 |
| Dietary notification | 1.00 | 0.97 – 1.03 | 0.76 |
| Age > 65 years | 0.95 | 0.83 – 1.06 | 0.39 |
| Man | 1.03 | 0.91 – 1.15 | 0.65 |
| White race | 1.33 | 1.14 – 1.53 | <0.001 |
| Baseline step count above mean | 1.69 | 1.50 – 1.89 | <0.01 |
| Step count 30 minutes prior to decision point | 1.64 | 1.58 – 1.71 | <0.01 |
| Time in study, days | 0.98 | 0.96 – 1.00 | 0.05 |

**Supplemental Table 5. Effect of activity or dietary notification on step count 60 minutes after a decision point.** Models adjusted for age (<65 versus >65), gender, race (White versus non-white), baseline mean daily step count (dichotomized on mean value), standardized step count 30 minutes before a decision point, and time (day in study). Results exponentiated for interpretability.

|  | **Estimate** | **95% Confidence Interval** | **P-value** |
| --- | --- | --- | --- |
| **Activity notification** | 1.01 | 0.99 – 1.04 | 0.33 |

**Supplemental Table 6: Impact of receiving an activity notification on step count 60-minutes after a decision point.** Results from sensitivity analysis in which we excluded data from participants with 0 or missing step count data in the three hours after a decision point. Results exponentiated for interpretability.

|  | **Estimate** | **95% Confidence Interval** |
| --- | --- | --- |
| Dietary notification | 0.93 | 0.83 – 1.04 |
| Age > 65 years | 1.21 | 0.84 – 1.59 |
| Man | 0.77 | 0.48 – 1.06 |
| White race | 1.01 | 0.62 – 1.41 |
| Baseline sodium intake above mean | 0.71 | 0.44 – 0.98 |
| Lower sodium choices 30 minutes prior to decision point | 0.94 | 0.55 – 1.32 |
| Time in study, days | 1.00 | 0.99 – 1.00 |

**Supplemental Table 7. Effect of dietary notifications on lower sodium food choices 24 hours after a decision point.** Analysis included only decision points at which participants received a dietary notification and no additional dietary notifications were sent in the subsequent 24-hours. These were contrasted to decision points at which no dietary notification was sent at that time or in the subsequent 24 hours. Models adjusted for age (<65 versus >65), gender, race (White versus non-white), baseline sodium intake (dichotomized on mean value), lower sodium food choices 30 minutes prior to a decision point, and time (days in study).


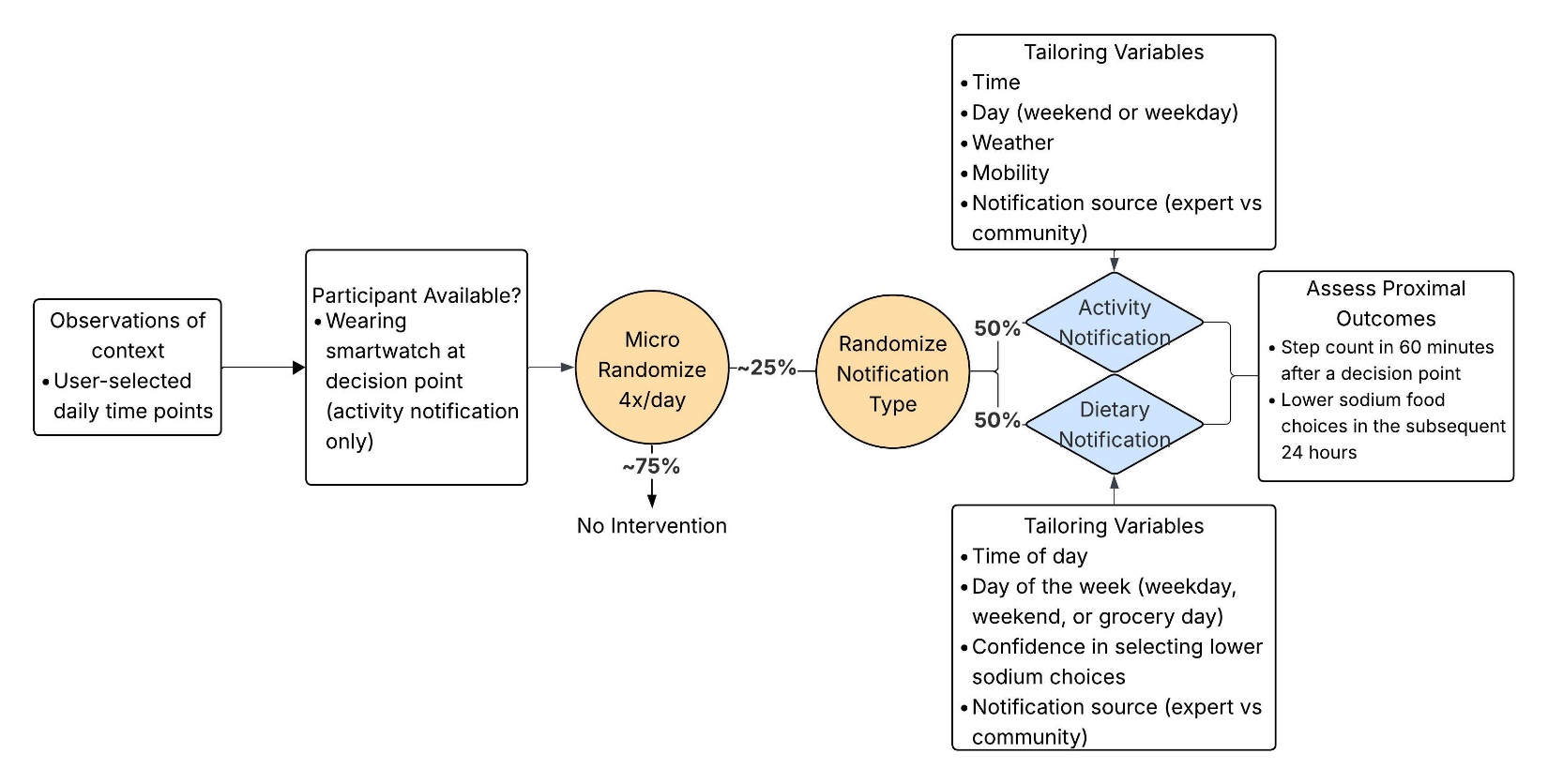


**Supplemental Figure 1: Micro-randomized Trial Design.** Notifications were designed to promote low-level physical activity and the selection of lower-sodium food choices. Activity notifications were tailored based on the time of day (i.e., morning, lunch, afternoon, evening), day of week (i.e., weekend versus weekday), weather, and mobility. Dietary notifications were tailored according to the time of day, day of the week (i.e., weekend, weekday, or grocery day), and participants’ confidence in selecting lower sodium food choices. Both notification types consisted of expert-generated and community-generated notifications, with the latter tailored based on participants’ site of enrollment (i.e., University of Michigan Health or Hamilton Community Health Network). Participants had a 25% probability of receiving a notification at each decision point, divided equally between activity and dietary notifications. The proximal outcome for activity notifications was step count 60 minutes after a decision point. For dietary notifications, the proximal outcome was self-reported lower sodium food choices in the mobile application within 24 hours of a decision point.
